# The 30 m land cover dataset for capturing land cover changes induced by ecological restoration from 1990 to 2022 on the Chinese Loess Plateau

**DOI:** 10.1038/s41597-025-04575-y

**Published:** 2025-02-12

**Authors:** Zhihui Wang, Xiaogang Shi, Shentang Dou, Miaomiao Cheng, Lulu Miao

**Affiliations:** 1 Key Laboratory of Soil and Water Conservation on the Loess Plateau of Ministry of Water Resources, Yellow River Institute of Hydraulic Research, Yellow River Conservancy Commission, Zhengzhou, China; 2 School of Social and Environmental Sustainability, University of Glasgow, Dumfries, UK

**Keywords:** Environmental sciences, Ecology

## Abstract

Continuous time-series of land cover is critical for attributing runoff, sediment and carbon changes on the Chinese Loess Plateau (CLP). However, current land cover products with annal temporal resolution lack spatial identification accuracy, particularly in capturing authentic changes of cropland, forest and grassland. To address these issues, a 30 m annual land cover dataset was proposed by the Yellow River Conservancy Commission (YRCC_LPLC) for the CLP from 1990 to 2022. Different levels of land cover were classified using different combinations of spectral, monthly and annual temporal and topographic features and Random Forest classifier. Compared to other land cover products (45.64%–73.38%), the accuracy of YRCC_LPLC has a better performance with an overall accuracy of 85.16%. The YRCC_LPLC is capable of capturing not only the explicit spatial variation but also the change direction and change time of land cover, especially for the most critical conversion of cropland into forest and grassland induced by implementation of Grain to Green Program on the CLP.

## Background & Summary

Land cover plays a crucial role in the earth system, serving as a vital link between the biosphere, atmosphere, and hydrosphere^1^. It is also fundamental for simulating land surface processes and serves as a key variable in ecological and hydrological models^2–4^. Currently, human activities have increasingly caused dramatic land cover changes, impacting on water cycles, air quality, energy balance and biogeochemical cycles, biodiversity and the provision of ecosystem services^5–7^. Consequently, obtaining precise and continuous time series of land cover data over a long-term period is critical for understanding climate and environmental dynamics^8^ and studying the interplay between human activities and global changes^9^. With the rapid economic development in China over recent decades, the country has encountered numerous environmental challenges, including desertification, sandstorms, soil erosion, and land degradation^10,11^. The Chinese Loess Plateau (CLP) is situated in northwestern China, spanning approximately 64,000 square kilometers. The mean annual precipitation across the CLP are between 300 mm and 600 mm, with the mean annual temperature ranging from 4 °C to 14 °C. This region is a typical arid and semi-arid environment where water scarcity has become a critical constraint on the regional socio-economic development and ecological progress. Historically, both climate change and excessive land cultivation have caused severe ecological degradation and water and soil erosion in the CLP^12–14^, leading to serious sedimentation and severe socioeconomic issues. To mitigate devastating environmental problems, the Chinese government has initiated several ecological restoration programs, including the ‘Three Norths’ Shelterbelt Program (TNSP)^15^ and the Grain to Green Program (GTGP)^16^. As a result, significant land cover and land use changes have been observed in the CLP since the GTGP began in 1999^17^, particularly in the loess hills and gully areas where there has been a notable decrease in cropland and an increase in forest and grassland^18,19^. Recently, it has been reported that land cover changes in the CLP contribute significantly to a marked reduction of runoff and sediment within the Yellow River Basin^20,21^, thus quantitatively assessing the impact of vegetation type changes on soil and water loss in the Loess Plateau has become a prominent research focus^22,23^. Consequently, land cover data has become essential for attributing the runoff and sediment reduction in tributaries and evaluating the ecological benefits of vegetation restoration efforts in the CLP.

There are several commonly used land cover products in the CLP, including the European Space Agency Climate Change Initiative (ESA-CCI) land cover product (300 m)^24^, the MCD12Q1 product (500 m)^25^, and the University of Maryland (UMD) land cover map (1 km)^26^. However, the spatial resolution of these products is too coarse to capture the highly heterogeneous land cover distribution caused by the fragmented terrain across the Loess Plateau. The free availability and accessibility of high-resolution Earth Observation (EO) data (e.g., Landsat)^27^ has facilitated fine-scale land cover monitoring on a large scale, leading to the development of many products with a resolution of 30 meters or higher. Some examples include the Finer Resolution Observation and Monitoring of Global Land Cover product (FROM-GLC) in 2010, 2015, and 2017^28^, GlobeLand30^29^ in 2000, 2010, 2020, annual China land cover dataset (CLCD)^30^ from 1990 to 2022, Global land-cover product with fine classification (GLC_FCS30)^31^ for every five years from 1985 to 2000, and in every year from 2001 to 2022. However, it has been reported that current available land cover products still lack classification and change detection accuracy, particularly in capturing authentic changes of cropland, forest and grassland induced by ecological restoration projects over the CLP^18,32^, which have severely affected the unveil of response mechanisms of eco-hydrological processes to underlying surface change in the CLP, and have significantly limited the accuracy of flood and sediment simulation and prediction in the Yellow River Basin^18^. To address these challenges, the Yellow River Conservancy Commission Loess Plateau Land cover (YRCC-LPLC) annual dataset from 1990 to 2022 was generated by YRCC. In this dataset, water bodies and snow/ice are directly from the CLCD product, whereas terrestrial areas were reclassified using the proposed method. Land cover samples were firstly collected using the combination of CLCD, Landsat time-series stack, high resolution satellite imagery and Google Earth images. Then the different specific remote sensing features were input into the Random Forest (RF) classifier to hierarchically classify land cover types. Subsequently, the accuracy of YRCC_LPLC was evaluated using testing samples, and further compared with the widely used global and national land cover products to demonstrate its advantages of detecting spatial and temporal land cover variations. Based on the YRCC_LPLC dataset, the long-term trends in land cover changes and conversions were detected across the CLP over the past three decades.

## Methods

### Satellite data

Landsat satellites have been providing 30-m global Earth Observation data, making them a widely recognized data source for high-resolution and large-scale land cover and land change mapping. Firstly, all available Landsat 5, 7 and 8 surface reflectance (SR) time-series stacks were collected from the T1_L2 (USGS Level 2, Collection 2, Tier 1) dataset, which contains 4 visible and near-infrared (VNIR) bands and 2 short-wave infrared (SWIR) bands processed to atmospherically corrected and orthorectified surface reflectance, in the Google Earth Engine (GEE) platform. Secondly, cloud and cloud shadow pixels in the SR were automatically detected using the program code of CF-mask algorithm^33^ in the GEE to remove the pixels contaminated by cloud and cloud shadow. In addition, ALOS Global Digital Surface Model “ALOS World 3D-30m (AW3D30)”, which is able to accurately capture topographic changes over Chinese mountainous area^34^, were used as the digital elevation model (DEM) to derive other topographic features, as shown in the Table 1.

**Table 1:** Detailed information about satellite data used in this study.

Satellite data	Spatial resolution	Temporal duration	Data source
Landsat5 TM	30 m	1990~2012	https://code.earthengine.google.com/
Landsat7 ETM+	30 m	1999~2003	https://code.earthengine.google.com/
Landsat8 OLI	30 m	2013~2020	https://code.earthengine.google.com/
AW3D30	30 m	—	https://www.eorc.jaxa.jp/ALOS/en/dataset/aw3d30/aw3d30_e.htm

### Land cover classification

#### Overall scheme

**Fig. 1:**
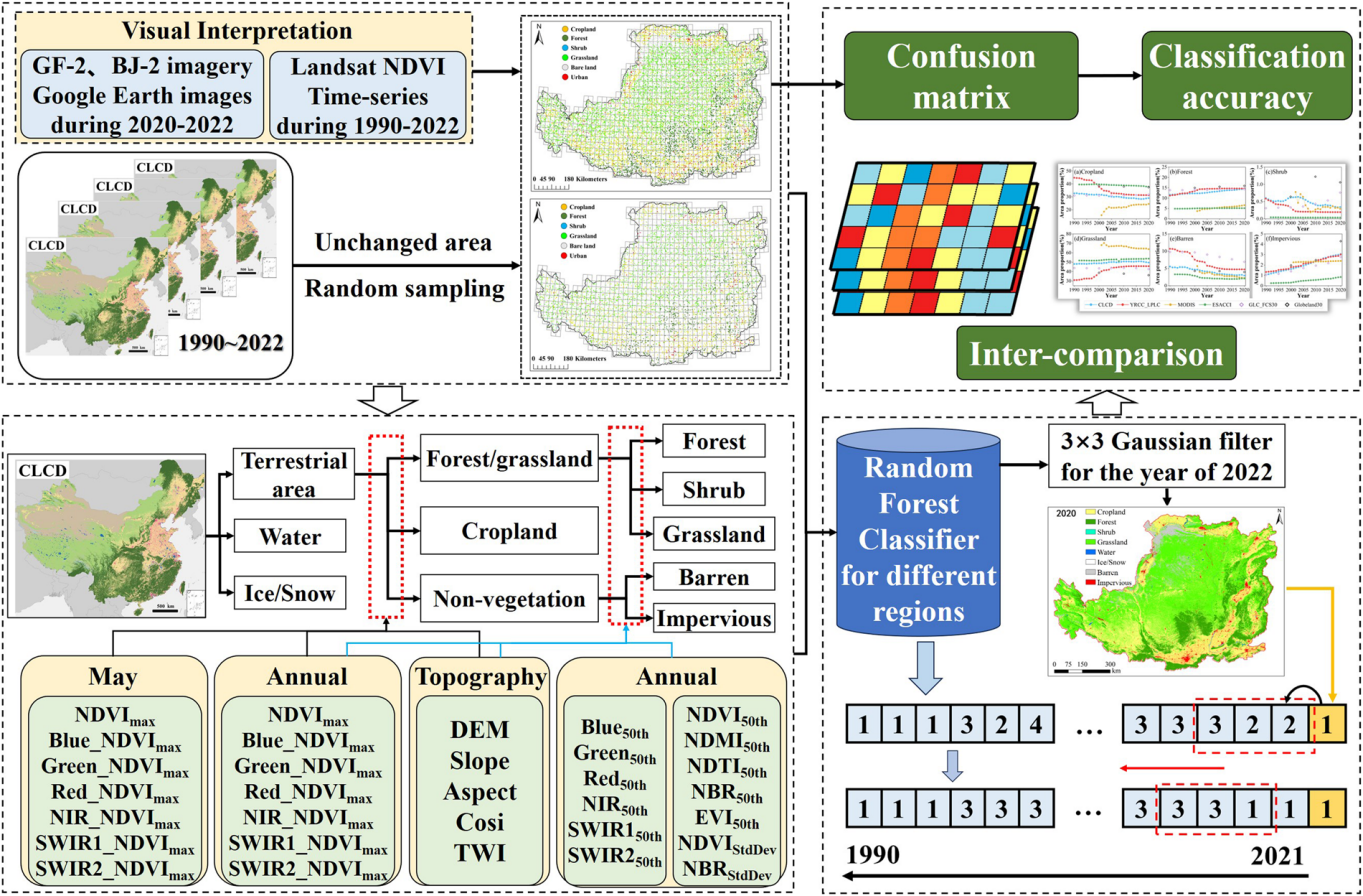
The flow chart to produce the annual land cover dataset on CLP. The numbers in the bottom right corner of the figure represent different land cover types (1:cropland, 2:forest, 3:shrub, 4:grassland).

This study aimed to generate the YRCC-LPLC dataset through a comprehensive processing workflow, which included the generation of training and testing samples, the development of input features for different land cover types, the RF classification model, spatial-temporal consistency validation, and accuracy evaluation along with the comparison between different land cover products (Fig. 1). The temporal composite metrics were obtained using the GEE platform providing long-term Earth observation data, and samples for land cover mapping were collected using the combination of current land cover products, high resolution imagery and GEE. Six land cover types including cropland, forest, shrub, grassland, barren, impervious were classified using the RF algorithm. The YRCC-LPLC’s accuracy was validated using independent test samples. Additionally, the YRCC-LPLC was compared with the widely used global land cover products.

#### Training and testing samples collection

**Fig. 2:**
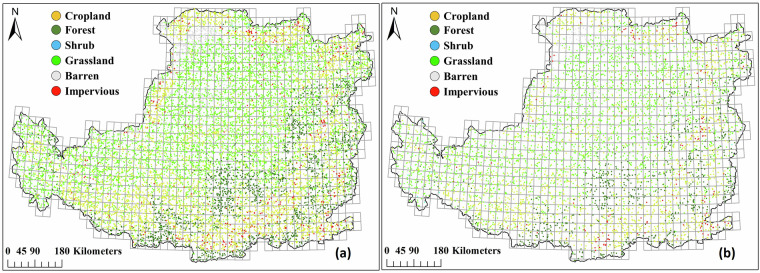
Spatial distribution of training samples (**a**) and testing samples (**b**) on the CLP.

For the supervised large-scale land cover mapping, the accurate and sufficient training samples are critically important^35^. Referring to the study of Yang and Huang^30^, the visual interpretation method and automatic sample extraction via existing LC products were both used to collect training samples in this study. Firstly, since annual CLCD achieved a higher overall accuracy than other global land cover products and has been widely used in various studies in China^30^, it was taken as a valuable source for collecting samples. The pixels without land cover change during 1990–2022 were extracted based on this dataset. Then, the CLP region was divided into 986 grids with 30 km sides, and 30 samples were randomly collected within each grid from pixels without land cover change in last step. In such a way, we obtained a total of 29,580 candidate samples of the CLP. Thirdly, if NDVI time-series curve of a candidate sample was stable (there is no significant change trend in annual 25^th^, 50^th^, 75^th^ percentile NDVI time-series), and its land cover type was the same as that visually interpretated using GF-2, BJ-2, Google Earth images from 2020 to 2022, this sample will be used to input classification model. For grid with less than 5 final samples, we visually interpretated some samples based on GF-2, BJ-2, Google Earth images and Landsat NDVI time-series trajectory in order to ensure uniform spatial distribution and diversity of all samples. It should be noted that we used Google Maps photo sphere to distinguish similar land covers (e.g. bare soil and dried grassland) when Google Earth images are not helpful for identifying land cover types. Finally, a total of 17,448 samples were finally determined from potential sample pool, and the training and testing samples at a ratio of 7:3 were illustrated in Fig. 2.

#### Input features for classification

The input features for classification were derived from spectral features and topographic characteristics. Different land cover types exhibit distinct reflectance characteristics across various spectral bands, allowing the use of band-specific reflectance to distinguish between them. To maximize the utility of Landsat data across all temporal phases, the 50^th^ percentile values of all available epochs were calculated for each spectral bands (Blue, Green, Red, NIR, SWIR1 and SWIR2) year by year. Due to spectral indexes can effectively enhance the difference among different vegetation types^36^, NDVI (Normalized Difference Vegetation index)^37^, NDMI (Normalized Difference Moisture Index)^38^, NDTI (Normalized Difference Tillage Index)^39^, NBR (Normalized Burnt Ratio)^40^ and EVI (Enhanced Vegetation index)^41^ were composited into the 50th percentiles using the annual composite method. Because the annual maximum NDVI (NDVI_max_) can effectively differentiate between vegetation and non-vegetation, as well as various vegetation types^30,42^, the SR of different spectral bands corresponding to the maximum NDVI (Red_NDVI_max_) were also used as the input features.

Given that the spectral characteristics of various land covers fluctuate throughout the year, the standard deviation (SD) of NDVI and NBR was also calculated to reflect the phenological information. It should be noted that based on prior knowledge about the spectral phenology characteristics of cropland and forest-grass vegetation on the Loess Plateau^18^, we used the monthly maximum NDVI in May and its corresponding SR values as input features to enhance the classification accuracy of cropland and forest-grass vegetation. Currently, numerous studies have shown that DEM and its derived variables, such as slope and aspect, are essential and significant auxiliary variables for land cover mapping^30,43^. Due to spatial distribution of vegetation types depends on local site condition determined by soil moisture, temperature and solar radiation in the hilly and gully areas, topographic wetness index (TWI)^44^ and illumination local angle (Cosi)^45^ also were considered to improve classification accuracy. Detailed feature descriptions were shown in the Table 2.

**Table 2:** Detailed description about input features in Random Forest for land cover mapping.

Features	Description
Blue_50th_ Green_50th_ Red_50th_ NIR_50th_ SWIR1_50th_ SWIR2_50th_	50^th^ percentile SR value for Blue, Green, Red, NIR, SWIR1 and SWIR2 band within a target year
NDVI_50th_ NDMI_50th_ NDTI_50th_ NBR_50th_ EVI_50th_	50^th^ percentile spectral indexes within a target year NDVI = (NIR-Red)/(NIR + Red) NDMI = (NIR-SWIR1)/(NIR + SWIR1) NDTI = (SWIR1- SWIR2)/(SWIR1 + SWIR2) NBR = (NIR- SWIR2)/(NIR + SWIR2) EVI = 2.5 × (NIR-Red)/(NIR + 6 × Red −7.5 × Blue + 1)
NDVI_max_	Annual maximum NDVI
	Monthly maximum NDVI in May
Blue_NDVI_max_ Green_NDVI_max_ Red_NDVI_max_ NIR_NDVI_max_ SWIR1_NDVI_max_ SWIR2_NDVI_max_	SR value of Blue, Green, Red, NIR, SWIR1 and SWIR2 band corresponding to annual maximum NDVI
	SR value of Blue, Green, Red, NIR, SWIR1 and SWIR2 band corresponding to monthly maximum NDVI in May
NDVI_StdDev_ NBRS_tdDev_	Annual standard deviation of NDVI and NBR
DEM	Elevation of land surface
Slope	The extent that a soil surface has an incline relative to the horizontal
Aspect	The direction a terrain surface faces in relation to compass directions
Cosi	${cosi}=\cos \theta \,\cos \alpha +\sin \alpha \,\sin \mathrm{\theta \; cos}\left({\varphi }_{s}-\beta \right)$, where $\theta$ is the solar zenith angle; ${\varphi }_{s}$ is the solar azimuth angle; $\alpha$ is the terrain slope; $\beta$ is the terrain aspect; $i$ is the local incidence angle.
TWI	$T=\mathrm{ln}\left(\frac{\alpha }{\tan \beta }\right)$, where $\beta$ is the terrain slope; $\alpha$ is the local upslope area draining through a certain point per unit contour length.

#### Classification and post-processing

To minimize classification errors for each land cover type to the greatest extent possible, we used a hierarchical classification strategy to classify terrestrial area of CLP, which could control the classification errors in different levels^18^, as illustrated in Fig. 1. The RF classifier is widely employed for large-scale land cover mapping^36^ because of its numerous advantages, including the capability to manage high-dimensional input features, tolerance to sampling errors, and robustness in the presence of missing data^46,47^. Therefore, the RF classifier was utilized to produce the YRCC-LPLC. Terrestrial area was firstly identified from CLCD, and then forest/grassland, cropland and non-vegetation were classified using the combination of NDVI_max_ in May and its corresponding RS, annual NDVI_max_ and its corresponding RS, and topographic features, Finally forest, shrub, grassland, barren and impervious were further classified based on annual NDVI_max_ and its corresponding SR, annual 50^th^ percentile composites in vegetation indexes and SR, annual SD of vegetation indexes and topographic features.

To ensure the consistency of the classification across spatial and temporal variations, post-classification processing workflow including spatial filter and temporal logical reasoning was individually used to refine the YRCC-LPLC data. Due to the samples used in this study were visually interpreted by high resolution imagery mostly acquired in 2022, the classification for 2022 using these samples must be the most accurate among 30 years. Firstly, A 2-D Gaussian low pass filter with 3 × 3 filtering window^48^ was applied into land cover map of 2022 to reduce some noise induced by misclassification. In the process of temporal filtering for land cover products, the land cover from 2022, which has higher confidence, should be used as a benchmark to filter the land cover data from earlier years. This ensures that the data from previous years are adjusted based on the more reliable classification from 2022. Therefore, we then considered the classification result of 2022 filtered by Gaussian low pass filter as benchmark, and temporal logical reasoning with a sliding 3-year window was employed from 2019 to 1990. Specifically, for year t, if the land cover labels for years t−2, t−1, and t within 3-year window were inconsistent, the label for year t would be revised into that of year t + 1; if the land cover labels for years t−2, t−1, and t were the same, the label for year t would remain unchanged.

#### Accuracy assessment

5234 testing samples (Fig. 2(b)) were employed to evaluate YRCC-LPLC, which are independent with the RF training samples. The accuracy of YRCC-LPLC was evaluated using confusion matrix which is a table used to evaluate the performance of a classification algorithm. Confusion matrix shows the number of correct and incorrect predictions made by the model, segmented by each class. It helps to visualize the accuracy of the model and identify where it is making errors. From the confusion matrix, various performance metrics including producer’s accuracy (PA), user’s accuracy (UA), overall accuracy (OA), and Kappa coefficient were derived. Since several land cover types in the study area have undergone significant changes, the F1 score was used to assess the classification accuracy of these specifical types that have experienced substantial changes. The F1 score reflects the balance between UA and PA, with a maximum value of 1 indicating the best accuracy and a minimum value of 0 indicating the worst^49,50^. The calculation formula is as follows:

1 $$F1=2\frac{{PA}\times {UA}}{({PA}+{UA})}\times 100 \% $$

For more comprehensive quality evaluation, we intercompared the YRCC-LPLC with four state-of-the-art global or national land cover products, including the MCD12Q1^25^, ESACCI^24^, Globeland30^51^, CLCD^30^ and GLCFCS30^31^, as demonstrated in the Table 3. To ensure comparability with the YRCC-LPLC, these four products were reclassified according to the YRCC-LPLC classification system to enable a more straightforward comparison. In addition, high-resolution google earth images were overlaid on different land cover products to evaluate their local classification performance.

**Table 3:** Detailed information of land cover products used for comparison with YRCC_LPLC.

Product	Resolution	Temporal duration	Data source
MCD12Q1	500 m	2000~2022	https://ladsweb.modaps.eosdis.nasa.gov/search/order/1/MCD12Q1
ESACCI	300 m	2000~2022	https://maps.elie.ucl.ac.be/CCI/viewer/
Globeland30	30 m	2000, 2010, 2020	http://www.globallandcover.com/
CLCD	30 m	1990~2022	https://zenodo.org/records/4417810
GLCFCS30	30 m	1990, 1995, 2000, 2001~2022	https://zenodo.org/records/8239305

## Data Records

**Fig. 3:**
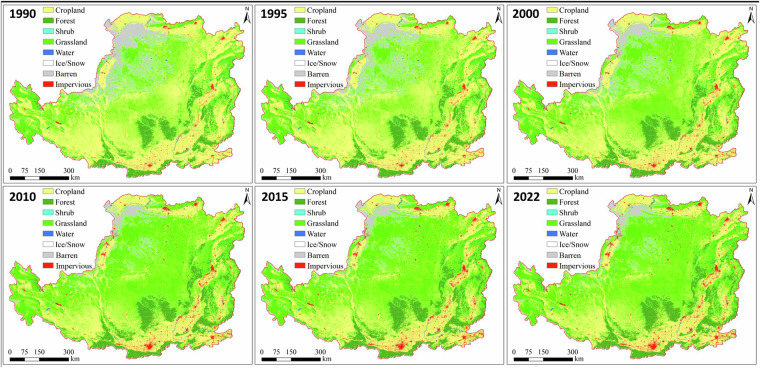
Land cover maps on the Loess Plateau in the 1990, 1995, 2000, 2010, 2015 and 2022.

**Fig. 4:**
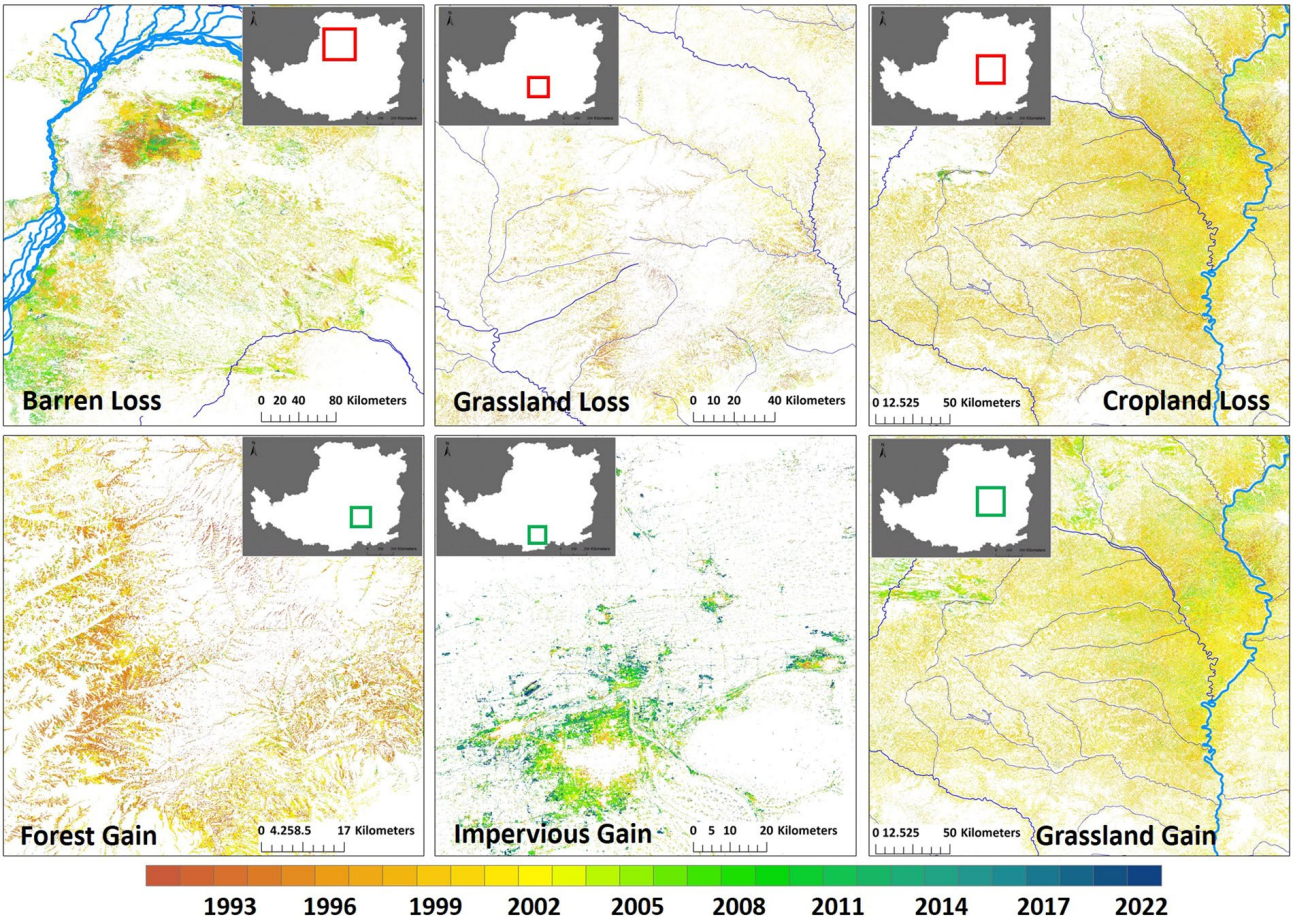
Changing time of main land covers in typical areas on the CLP from 1990 to 2022.

The annual land cover classification data from 1990 to 2022 is available at Zenodo (10.5281/zenodo.10225564)^52^. The ZIP file labeled by different year (e.g. 2020_landcover.zip) contains Geotiff file with a spatial reference of the WGS84 coordinate system. The accompanying Excel file, “landcover_classificationsystem.xlsx”, provides a comprehensive description of the land cover classification system utilized in the dataset. Different integers correspond to various types of land cover: 1 cropland; 2 forest; 3 shrub; 4 grassland; 5 water; 6 ice/snow; 7 barren; and 8 impervious. This classification system is similar to that of CLCD^30^ and can be easily reclassified to align with the FAO (Food and Agriculture Organization) classification system. The Geotiff data can be imported into standard remote sensing processing software and geographical information system software (e.g., ENVI and ArcGIS), and can be easily read by MATLAB, IDL, Python, etc. Figure 3 shows the land cover maps of 1990, 1995, 2000, 2010, 2015, 2022. Figure 4 shows the change time of specific land cover derived from this dataset.

## Technical Validation

### Accuracy assessment of YRCC_LPLC

The classification accuracy of different land cover product was first assessed by 5234 testing samples for each year, and the multi-year average of accuracy evaluation metrics for each land cover product was calculated respectively, as illustrated in Table 4. It should be noted that due to the difficulty in obtaining accurate information of changed land cover (including change time, land cover before change and land cover after change), we only obtained the samples of unchanged land cover. Therefore, the assessment using these samples represents accuracy in characterizing spatial distribution of land cover, whereas the accuracy of temporal change detection cannot be directly assessed.

**Table 4:** Comparison of multi-year average classification accuracy based on testing samples for YRCC_LPLC, CLCD, GLC_FCS30, ESACCI and MCD12Q1.

Product	Metric	Cropland	Forest	Shrub	Grassland	Barren	Impervious	OA (%)	Kappa
YRCC_LPLC	PA(%)	88.12	73.12	31.24	82.78	93.45	89.44	85.16	0.89
	UA(%)	92.79	78.65	26.78	91.47	78.63	78.15		
	F1(%)	90.39	75.78	28.84	86.91	85.40	83.41		
CLCD	PA(%)	64.52	73.46	30.14	61.83	82.23	84.11	73.27	0.78
	UA(%)	75.43	68.15	16.49	72.74	73.49	82.94		
	F1(%)	69.55	70.71	21.32	66.84	77.61	83.52		
GLC_FCS30	PA(%)	73.56	64.46	23.89	64.40	92.41	82.50	70.38	0.72
	UA(%)	48.75	73.50	7.41	77.87	74.85	79.30		
	F1(%)	58.64	68.68	11.31	70.50	82.71	80.87		
Globeland30	PA(%)	83.41	59.80	15.38	48.67	89.71	63.79	65.86	0.63
	UA(%)	52.27	77.30	2.50	75.18	53.73	53.47		
	F1(%)	64.27	67.43	4.30	59.09	67.21	58.18		
ESACCI	PA(%)	42.81	61.54	23.56	71.36	62.76	59.43	57.16	0.51
	UA(%)	74.39	68.30	12.71	32.06	80.41	40.22		
	F1(%)	54.35	64.74	16.51	44.24	70.50	47.97		
MCD12Q1	PA(%)	35.76	39.74	0.00	78.42	52.73	45.45	45.64	0.44
	UA(%)	69.82	62.54	0.00	21.26	62.33	39.41		
	F1(%)	47.30	48.60	0.00	33.45	57.13	42.22		

Overall, the accuracy of YRCC_LPLC has a better performance with OA of 85.16% compared to the CLCD (73.27%), the GLC_FCS30 (70.38%), the Globeland30 (65.86%) the ESACCI (57.16%) and the MCD12Q1 (45.64%), respectively, as shown in Table 4. Specifically, cropland had the highest average F1 score at 90.39%, followed by the grassland (86.91), barren (85.4%)and impervious (83.41) classes. Both grassland and cropland showed relatively high accuracy, with mean F1 scores exceeding 86%. Although YRCC_LPLC didn’t always have the highest UA or PA compared to other products, it attained the highest F1 scores across nearly all land cover types. For the land covers with a relatively large proportion in area, such as cropland, forest and grassland, CLCD and GLC_FSC30 exhibited higher F1 scores with respect to the Globeland30, ESACCI and MCD12Q1, whereas YRCC_LPLC demonstrated higher F1 score than CLCD and GLC_FCS30 across most land cover types, with particularly notable improvements in cropland and grassland, which underwent the most significant changes in the CLP.

### Comparison with other landcover products

#### Spatial distribution of land covers

**Fig. 5:**
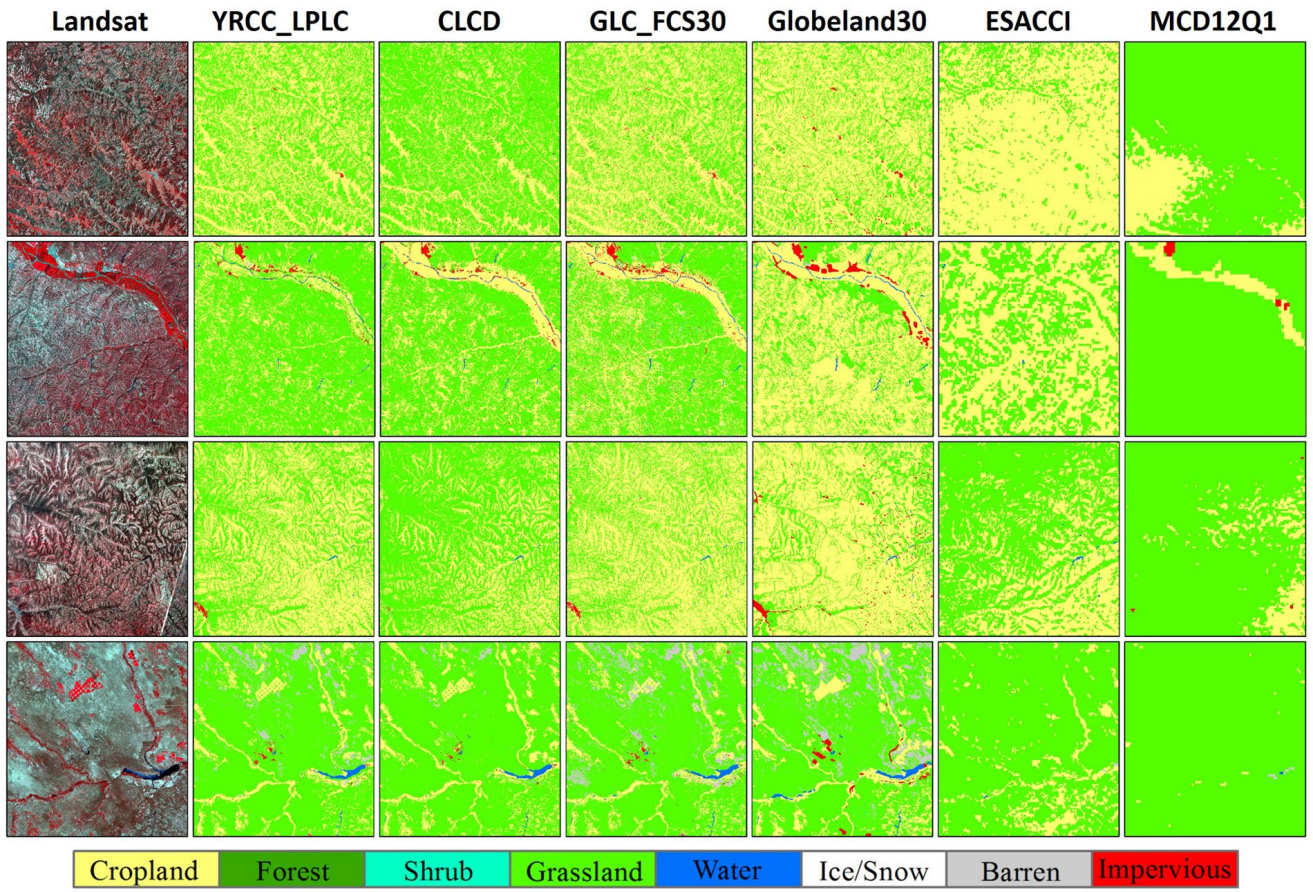
Comparison between YRCC_LPLC and CLCD, GLC_FCS30, Globeland30, ESACCI and MCD12Q1 for the year of 2020.

Except for the quantitative assessment, four regions and their local enlargements, covering various climate and landscape environment, were selected to directly illustrate the performance of each land-cover product in Fig. 5. The MCD12Q1 completely fails to capture the spatial heterogeneity of land cover, classifying nearly all areas as grassland. In contrast, the ESACCI can reflect the spatial distribution of land cover on a large scale, but it still exhibits noticeable classification errors. From the perspective of land cover diversity, it was obvious that the YRCC_LPLC, CLCD, Globeland30 and GLC_FCS30 had significant advantages over the other two products. Although Globeland30 can depict a more detailed spatial distribution of land cover, it over-identifies cropland and fails to identify the forest and grass vegetation derived from cropland retirement. The overall patterns of land cover types of GLC_FCS30, CLCD, and YRCC_LPLC are similar, however, there are still noticeable differences in the spatial distribution of various land cover types between them.

**Fig. 6:**
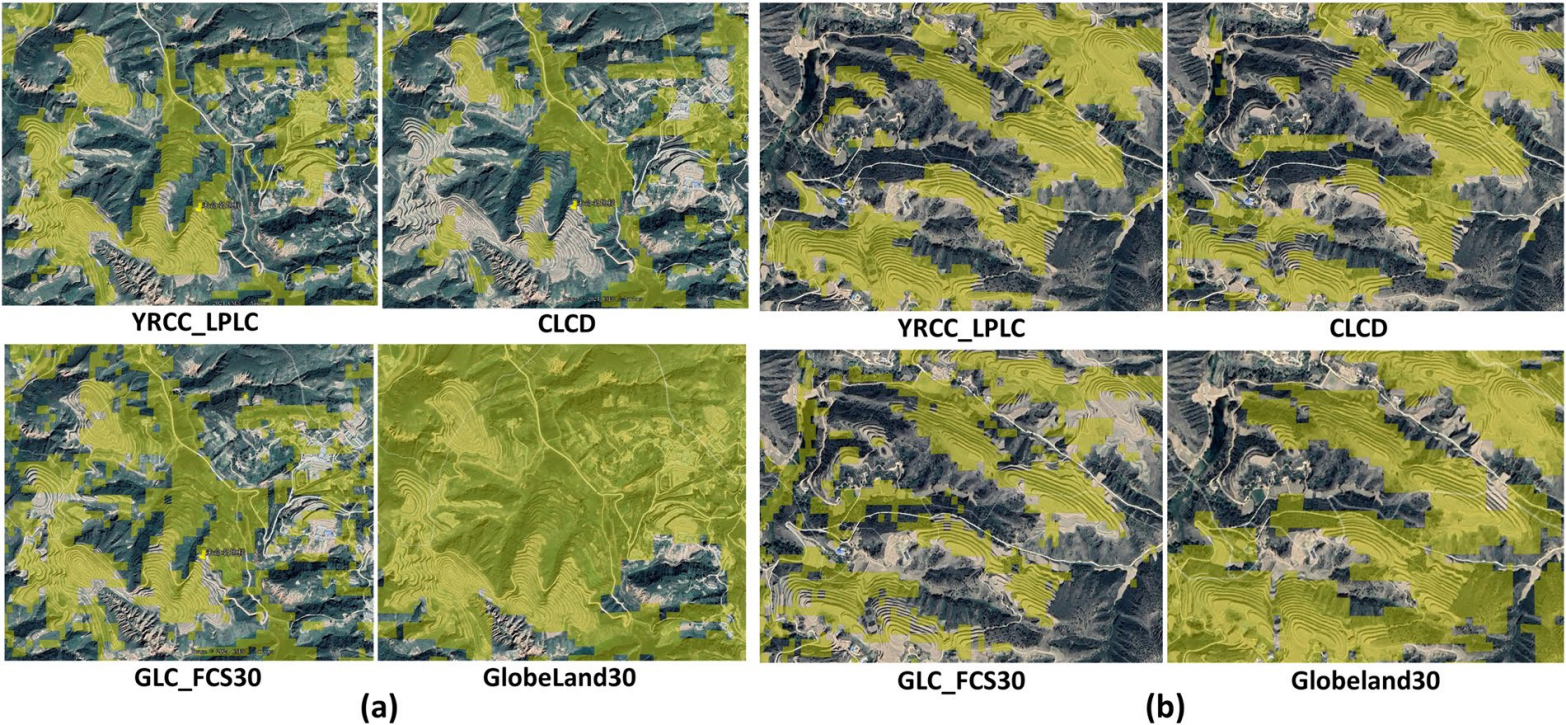
Cropland of CLCD, GLC_FCS30, YRCC_LPLC and Globeland30 in 2020 was overlayed on the Google Earth imagery acquired on 28^th^ Aug. 2020. (**a**) 37°49′35.96″N 109°52′51.39″E, (**b**) 37°48′13.77″N 109°53′58.46″E. Highlighted areas in yellow represent the classified cropland.

Given the combined effects of climate conditions and human activities on crop growth, along with its fragmented distribution and rugged terrain, accurately classifying cropland is quite challenging. Consequently, we chose two cropland areas in typical hilly regions for comparative analysis. On Google Earth imagery (2020-8-28), the extent of terraced fields is clearly visible. Ground surveys and prior knowledge confirm that crops such as corn and potatoes are grown on these terraced fields, allowing them to be identified as cropland. As shown in Fig. 6, Globeland30 classifies a significant amount of forest and grass vegetation as cropland, and both CLCD and GLC_FCS30 exhibit notable omission and misclassification issues. In contrast, YRCC_LPLC demonstrates a significantly superior performance in cropland classification compared to the other products, indicating that it can accurately depict the fine-scale spatial variations of different land cover types.

#### Temporal change of land covers

**Fig. 7:**
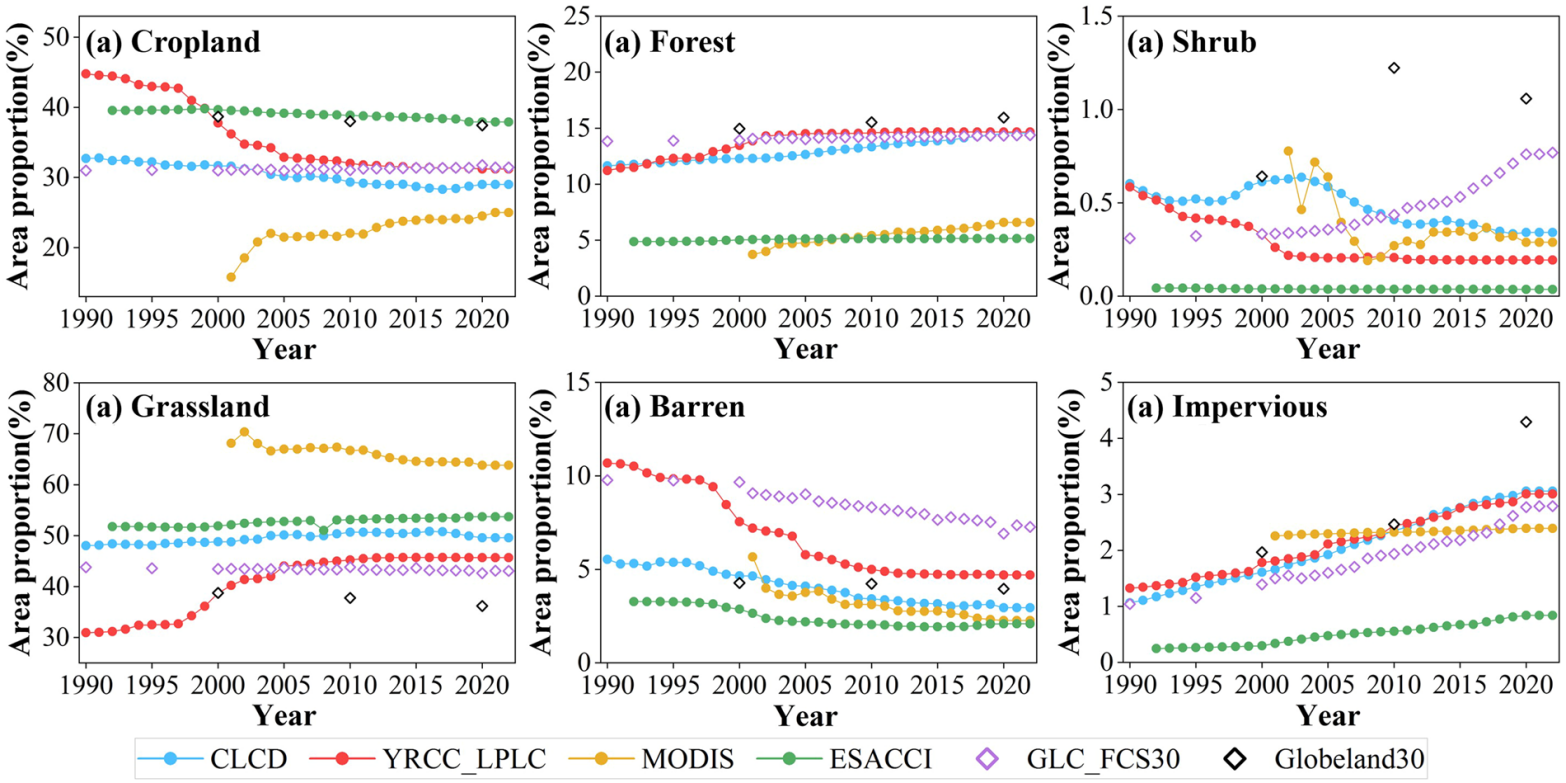
Temporal changes in area of different land covers on CLP from 1990 to 2022.

Based on different land cover products, we analyzed the dynamic changes in the area proportions of various land cover types from 1990 to 2022. As shown clearly in Fig. 7, the changes of cropland, grassland, and barren land in YRCC_LPLC are more pronounced compared to other products. Over 32 years, the proportion of cropland in YRCC_LPLC decreased by 13.58% of the whole CLP, whereas the cropland proportion in CLCD decreased by only 3.69%. The proportions in Globeland30 and ESACCI remained relatively stable, while MCD12Q1 even showed an increasing trend in cropland area. Similarly, the proportion of grassland in YRCC_LPLC increased by 14.73%, while ESACCI and CLCD showed only slight increases of 1.94% and 1.55%, with relatively high values. GLC_FCS30’s grassland area remained largely unchanged, and MCD12Q1 and Globeland30 even exhibited a decreasing trend in grassland area. For barren land, YRCC_LPLC showed a reduction of 5.98% over 30 years, whereas CLCD, GLC_FCS30, MCD12Q1, and ESACCI showed slight decreases of 2.58%, 2.94%, 3.4%, and 1.27%, respectively. The barren land proportion in Globeland30 remained essentially unchanged. YRCC_LPLC also demonstrated some correlation with other products for some specific periods. For instance, after 2010, YRCC_LPLC’s cropland area closely aligned with GLC_FCS30 and CLCD, and in 2000, it was more similar to Globeland30. The grassland area in YRCC_LPLC after 2005 closely matched GLC_FCS30, and the barren land area after 2010 aligned with Globeland30. Shrubland exhibited a similar decreasing trend found in CLCD. These findings indicate that other products only reflected slight change of cropland, grassland and barren land that should be changed due to extensive implementation of GTGP and soil and water conservation efforts^49^. In contrast, the YRCC_LPLC can accurately captured these obvious land cover changes over the CLP during 35 years.

**Fig. 8:**
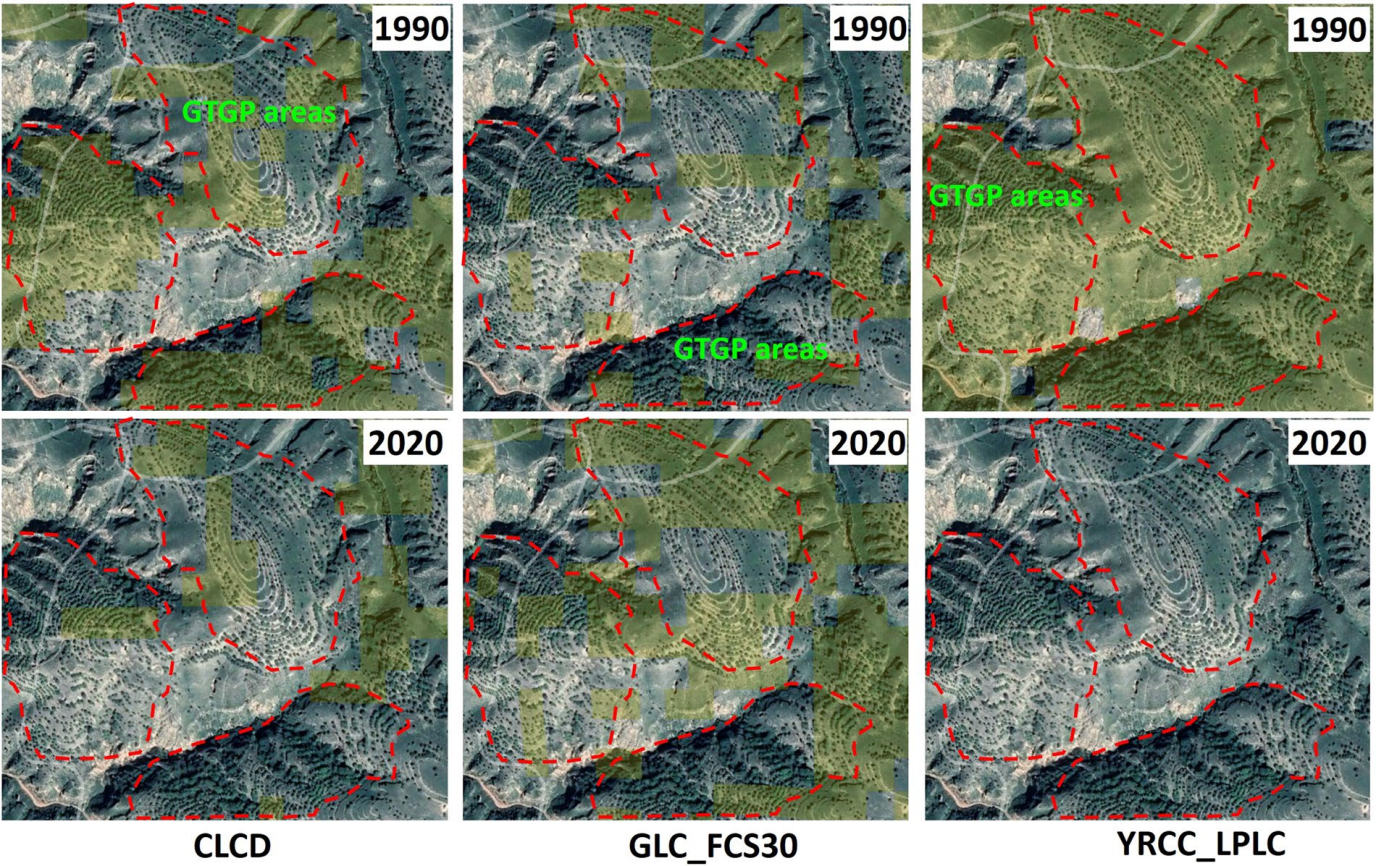
Cropland of CLCD, GLC_FCS30 and YRCC_LPLC for 1990 and 2020 was overlayed on the Google Earth imagery acquired on 28^th^ Aug. 2020 (37°49′35.96″N 109°52′51.39″E). Highlighted areas in yellow represent the classified cropland. Dashed red polygon represents typical GTGP area.

Additionally, we selected two 30-m products covering 1990 and 2020 (CLCD and GLC_FCS30) for a detailed local comparison with YRCC_LPLC to evaluate their accuracy in detecting cropland loss in a typical area of land conversion from cropland to forest and grassland. Three typical and known GTGP areas (red polygon) were displayed in the Google Earth imagery (2020-8-28), as shown in the Fig. 8. From the distinct texture features of terraces and prior knowledge about cropland cultivation in this area, we can safely conclude that this area used to be cropland during the 1990s. In addition, the imagery also shows scattered tree canopies and large areas of grassland within the terraces, indicating that this area has been abandoned and reforested due to the implementation of GTFP. However, CLCD and GLC_FCS30 identified only a small portion of the farmland in 1990, and showed little change in the cropland by 2020. In contrast, YRCC_LPLC accurately identified the entire farmland area in 1990 and detected a significant loss of cropland by 2020 (Fig. 8). This demonstrates that YRCC_LPLC has a very high accuracy in detecting main land cover changes on the CLP.

#### Changed and unchanged land covers

**Fig. 9:**
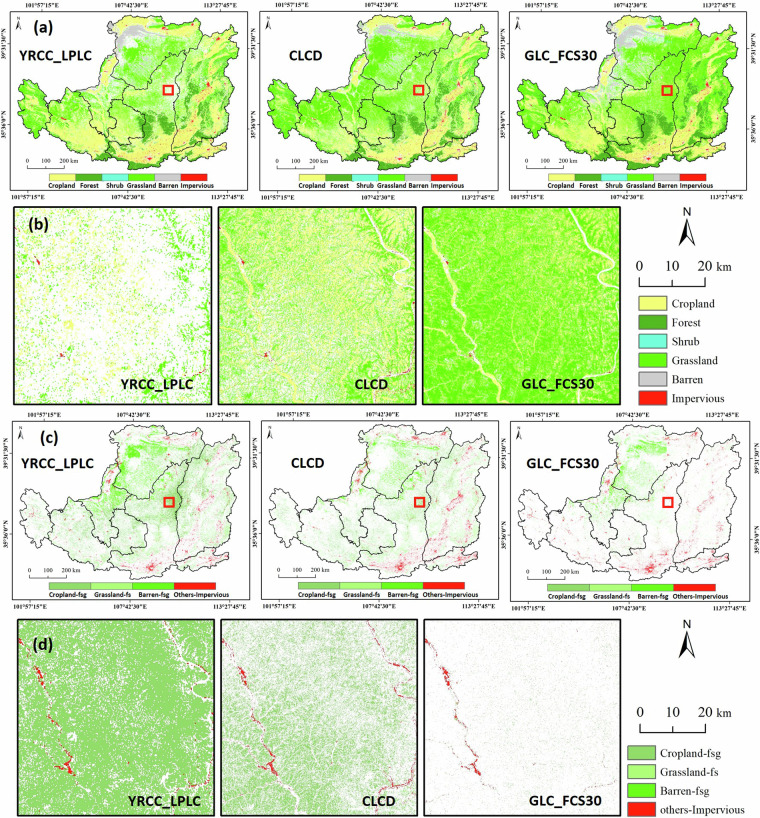
Distribution of unchanged land covers (**a,b**) (cropland, forest, shrub, grassland, barren and impervious), and changed land covers (**c,d**) (C-Fsg, G-Fs, B-Fsg and O-I). The red rectangle in the a and c represent a specific region shown in b and d, respectively.

**Fig. 10:**
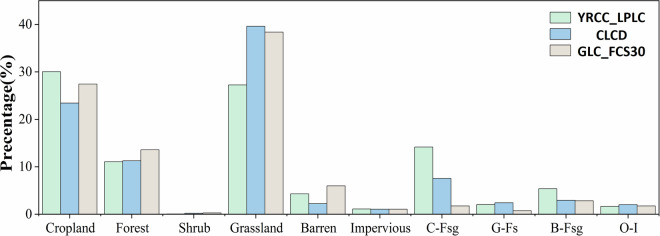
The area ratio of different unchanged and changed land covers from 1990 to 2022 for different land cover products.

Two 30-m products covering 1990 and 2022 (CLCD and GLC_FCS30) for a comparison with YRCC_LPLC to evaluate their accuracy in detecting unchanged and changed land covers. Figure 9 depict the spatial distribution of six unchanged land cover types including cropland, forest, shrubland, grassland, barren land, and impervious surfaces and four types of land cover changes including cropland to forest and grassland (C-Fsg), grassland to forest and shrubland (G-Fs), barren land to forest and grassland (B-Fsg), and other types to impervious surfaces (O-I). Figure 9(b–d) is the local zoomed-in image of changed land cover land unchanged land cover in a typical GFGP area. From the image, we can observe that cropland and grassland have undergone significant changes in the YRCC_LPLC, while the CLCD shows less change in land cover. Additionally, there is little change in land cover for GLC_FCS30. The statistical results for the area proportions of these different types are shown in Fig. 10. For the unchanged types, YRCC_LPLC has the highest proportion of cropland, accounting for 30%, followed by grassland at 27.3%, however, in CLCD and GLC_FCS30, the proportion of grassland is greater than that of cropland. For the changed types, the largest proportion in YRCC_LPLC is C-Fsg, making up 14.2%. As shown in the Fig. 9(c), C-Fsg was mostly distributed in the primary sediment-producing area in Northern Shaanxi region. B-Fsg was mainly located in the Mu Us Sandy Land and Kubuqi Desert, which accounts for 5.4% of the whole CLP. Obviously, it can be seen that the area proportions of these changed land covers are significantly higher than those in CLCD and GLC_FCS30. Overall, it can be inferred that land cover changes derived from YRCC_LPLC are more reliable in the context of implementing the GFGP over 30 years than those derived from CLCD and GLC_FCS30.

#### Chang year of cropland converting to forest and grassland

**Fig. 11:**
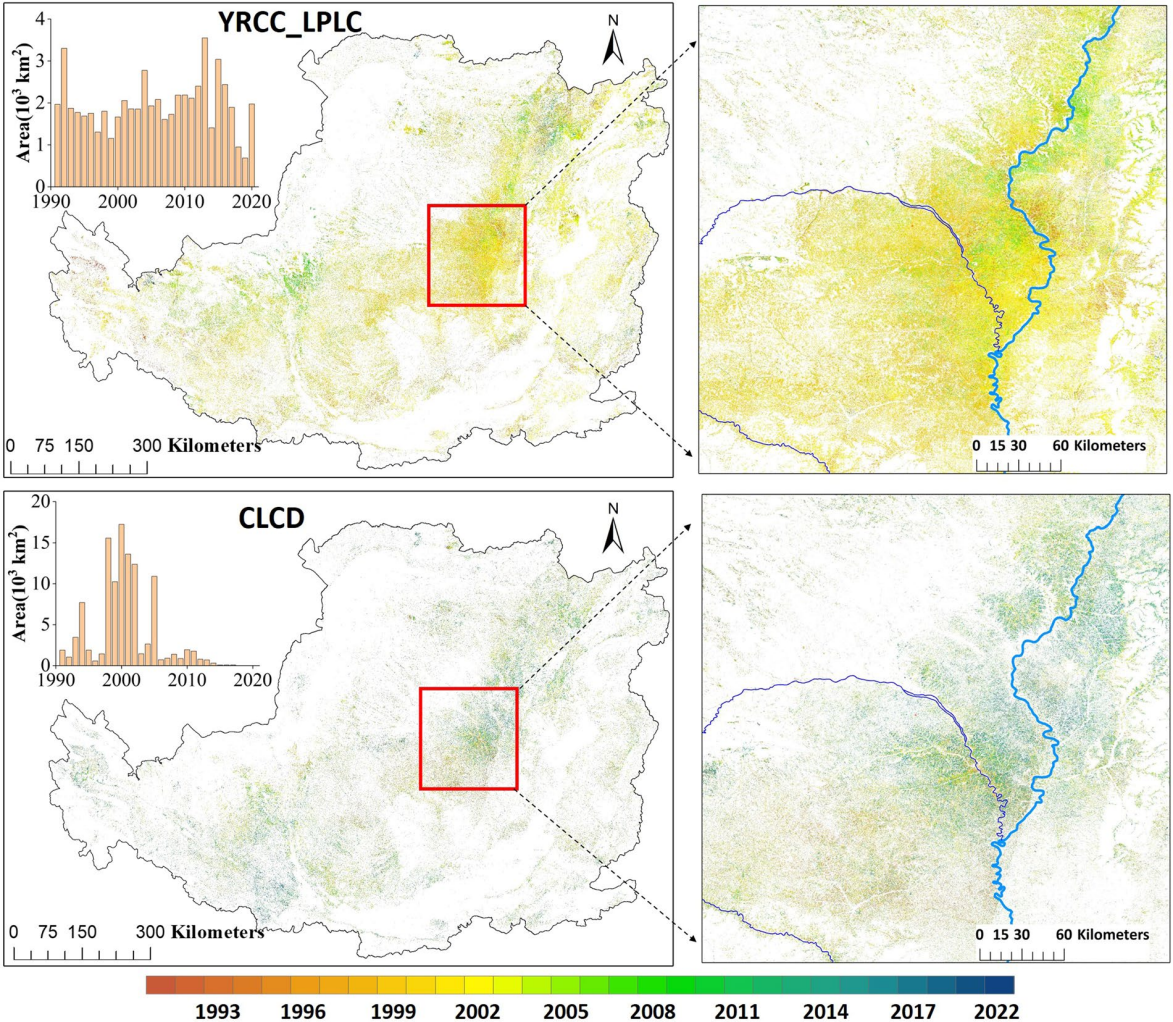
The time of cropland converted into forest and grassland derived from YRCC_LPLC and CLCD.

30-m annual land cover from 1990 to 2022 of CLCD was selected for a comparison with YRCC_LPLC to evaluate their accuracy in detecting change time of cropland converted into forest and grassland. For YRCC_LPLC, if the land cover in year t-1 is classified as cropland, and in year t it is classified as forest, grassland, or shrubland, then year t is defined as the change year. For CLCD, we utilized the abandoned cropland change detection method proposed by Zhang *et al*.^50^, and implemented an IDL program to automatically extract change year for each pixel. Figure 11 shows that the change years derived from YRCC_LPLC are primarily concentrated between 1998 and 2005, which aligns closely with the implementation period of China’s GTGP. Additionally, the areas of detected change largely correspond to the regions where GTGP was carried out. In contrast, the change years derived from CLCD are evenly spread across the period from 1991 to 2019, with most changes in the GTGP regions occurring after 2008, which clearly does not match the actual implementation timeline of GTGP. This finding also suggests that YRCC_LPLC offers superior accuracy in detecting land cover changes, particularly in identifying the critical land cover conversion of cropland into forests and grasslands on the Loess Plateau, compared to other existing products.

## Data Availability

The source code used the Python and IDL language. The source code contains four sections: changetime_class_filter.pro, detect_changetime.pro, clcd_landcover_change_time2.pro, RFClassify.py. The source code can be downloaded at https://github.com/wzh8588/YRCC_LPLC.
